# Combining various acupuncture therapies with multimodal analgesia to enhance postoperative pain management following total knee arthroplasty: a network meta-analysis of randomized controlled trials

**DOI:** 10.3389/fneur.2024.1361037

**Published:** 2024-03-18

**Authors:** Ningning Liu, Gaihong Liu, Xiaoli Chang, Yingxue Xu, Yi Hou, Dongbin Zhang, Lianzhu Wang, Shaozong Chen

**Affiliations:** ^1^Affiliated Hospital of Shandong University of Traditional Chinese Medicine, Jinan, Shandong, China; ^2^School of Acupuncture-Moxibustion and Tuina, Shandong University of Traditional Chinese Medicine, Jinan, Shandong, China; ^3^Research Institute of Acupuncture and Moxibustion, Shandong University of Traditional Chinese Medicine, Jinan, Shandong, China

**Keywords:** acupuncture therapy, total knee arthroplasty (TKA), pain management, alternative therapies, network meta-analysis (NMA), randomized controlled trials

## Abstract

**Objective:**

This study aims to evaluate the efficacy and safety of various acupuncture treatments in conjunction with multimodal analgesia (MA) for managing postoperative pain and improving knee function in patients undergoing total knee arthroplasty (TKA), based on the findings from clinical research indicating the potential benefits of acupuncture-related therapies in this context.

**Methods:**

We searched Web of Science, PubMed, SCI-hub, Embase, Cochrane Library, China Biology Medicine (CBM), China National Knowledge Infrastructure (CNKI), Wanfang Data, and Chinese Scientific Journal Database (VIP) to collect randomized controlled trials of acupuncture-related therapies for post-TKA pain. After independent screening and data extraction, the quality of the included literature was evaluated. The potential for bias in the studies incorporated in the analysis was assessed according to the guidelines outlined in the Cochrane Handbook 5.1. Network meta-analysis (NMA) was conducted using RevMan 5.4 and Stata 16.0 software, with primary outcome measures including visual analog scale (VAS), pain pressure threshold (PPT), hospital for special surgery knee score (HSS), and knee joint range of motion (ROM). Furthermore, the interventions were ranked based on the SUCRA value.

**Results:**

We conducted an analysis of 41 qualifying studies encompassing 3,003 patients, examining the efficacy of four acupuncture therapies (acupuncture ACU, electroacupuncture EA, transcutaneous electrical acupoint stimulation TEAS, and auricular acupoint therapy AAT) in conjunction with multimodal analgesia (MA) and MA alone. The VAS results showed no significant difference in efficacy among the five interventions for VAS-3 score. However, TEAS+MA (SMD: 0.67; 95%CI: 0.01, 1.32) was more effective than MA alone for VAS-7 score. There was no significant difference in PPT score among the three interventions. ACU + MA (SMD: 6.45; 95%CI: 3.30, 9.60), EA + MA (SMD: 4.89; 95%CI: 1.46, 8.32), and TEAS+MA (SMD: 5.31; 95%CI: 0.85, 9.78) were found to be more effective than MA alone for HSS score. For ROM score, ACU + MA was more efficacious than EA + MA, TEAS+MA, and AAT + MA, MA. Regarding the incidence of postoperative adverse reactions, nausea and vomiting were more prevalent after using only MA. Additionally, the incidence of postoperative dizziness and drowsiness following ACU + MA (OR = 4.98; 95%CI: 1.01, 24.42) was observed to be higher compared to that after AAT + MA intervention. Similarly, the occurrence of dizziness and drowsiness after MA was found to be significantly higher compared to the following interventions: TEAS+MA (OR = 0.36; 95%CI: 0.18, 0.70) and AAT + MA (OR = 0.20; 95%CI: 0.08, 0.50). The SUCRA ranking indicated that ACU + MA, EA + MA, TEAS+MA, and AAT + MA displayed superior SUCRA scores for each outcome index, respectively.

**Conclusion:**

For the clinical treatment of post-TKA pain, acupuncture-related therapies can be selected as a complementary and alternative therapy. EA + MA and TEAS+MA demonstrate superior efficacy in alleviating postoperative pain among TKA patients. ACU + MA is the optimal choice for promoting postoperative knee joint function recovery in TKA patients. AAT + MA is recommended for preventing postoperative adverse reactions.

**Systematic review registration:**

https://www.crd.york.ac.uk/, identifier (CRD42023492859).

## 1 Background

The incidence of knee osteoarthritis (KOA) has significantly risen due to the acceleration of population aging and the increasing prevalence of obesity and overweight individuals. Data from the China Health and Retirement Longitudinal Study (CHARLS) database indicate that symptomatic knee osteoarthritis now affects 13.7 and 10.8% of individuals in southwest and northwest China, respectively (1). TKA, a well-established and effective reconstructive treatment, has demonstrated long-term efficacy in managing severe multi-compartmental knee osteoarthritis with deformity. When compared to basic treatment, pharmacological intervention, and reparative approaches for KOA, TKA demonstrates significant improvements in pain relief and joint function (2), Western Ontario and McMaster Universities Osteoarthritis Index (WOMAC) scores, hospital for special surgery knee score (HSS), and overall quality of life during long-term follow-up.

The presence of pain after TKA is a significant factor that affects the outcomes of patient rehabilitation. According to the International Association for the Study of Pain (IASP) (3), pain is defined as an unpleasant sensory and emotional experience that is linked to actual or potential tissue damage and is influenced by biological, psychological, and social factors to varying extents. Pain can easily result in the development of depression, anger, and anxiety, which is why it is considered as the fifth vital sign (4). In contrast to total hip arthroplasty, it is imperative to incorporate high-intensity knee flexion and extension training after TKA to enhance functional activity and prevent complications such as postoperative knee stiffness. However, it is important to note that early functional exercise may potentially worsen postoperative pain. The relationship between postoperative pain and knee joint rehabilitation is reciprocal (5). The implementation of enhanced recovery after surgery (ERAS) in perioperative management has made it essential to effectively manage postoperative pain and knee swelling as an integral aspect of early rehabilitation (6).

Previous research has established that high levels of sedentary behavior put people with musculoskeletal conditions (such as osteoarthritis) at elevated risk for reduced physical function, increased physical frailty and blood pressure, and may increase mortality (7). However, physical activity provides a multitude of health advantages, encompassing the prevention and management of various ailments, such as hypertension, stroke, obesity, diabetes, and mental health disorders, such as anxiety and depression (8). Consequently, the World Health Organization has initiated the Global Action Plan on Physical Activity 2018–2030, with the objective of augmenting physical activity levels by 15% before 2030 (9). Regrettably, a concerning observation is that individuals undergoing total knee arthroplasty (TKA) often exhibit limited engagement in physical activity post-surgery (10), primarily due to their obesity that was present preoperatively. Merely, 5% of TKA patients adhere to national physical activity guidelines (11), which put them at increased risk of cardiovascular disease and cancer (12). The studies (13) have demonstrated that augmenting physical activity can effectively enhance gait function following TKA, with a realistic and appropriate target of approximately 3,000 steps per day for postoperative TKA patients. This suggests the attainability of heightened postoperative gait function. Additionally, another study (14) has revealed that initiating ambulation and engaging in 15–30 min of walking twice daily, commencing on the initial day after surgery, can significantly diminish the occurrence of thromboembolic complications subsequent to TKA. Consequently, it is imperative to augment physical activity following TKA.

The implementation of multimodal analgesia in the perioperative pain management of TKA has gradually gained traction (15). Within the ERAS framework, multimodal analgesia not only plays a central role but also demonstrates a strong interconnection with other rehabilitation measures. The primary goal of perioperative pain management has evolved beyond solely providing pain relief to encompass facilitating the prompt recovery of patients as a whole (16). Moreover, when incorporating multimodal analgesia, careful attention must be paid to its effects on the entire ERAS system (17). Multimodal analgesia encompasses a range of interventions, both non-pharmacological and pharmacological, that aim to manage pain. Non-pharmacological interventions include acupuncture, moxibustion, massage, and psychological guidance. Pharmacological interventions involve the use of various drugs and delivery routes, such as traditional Chinese medicine, patient-controlled analgesia (PCA), regional nerve block, intravenous/oral administration of non-steroidal anti-inflammatory drugs (NSAIDs) and opioids, as well as periarticular multimodal drug injection (PMI) (18).

The administration of femoral nerve block, iliac fascia nerve block, adductor canal block, and sciatic nerve block to alleviate postoperative pain in TKA patients may lead to varying degrees of muscle strength decline in the quadriceps femoris, biceps femoris, gastrocnemius (19), and other related muscles. This decline in muscle strength is not beneficial for the isometric contraction rehabilitation exercise of these muscles following TKA. In multimodal analgesia, analgesics are commonly administered in a stepwise manner. For the management of mild pain, oral non-steroidal anti-inflammatory drugs (NSAIDs) and weak opioids, such as celecoxib, flurbiprofen axetil, and nalbuphine (20), are commonly prescribed. On the other hand, for moderate-to-severe pain, strong opioids or narcotic analgesics, such as morphine, butorphanol, and fentanyl (21), are typically utilized. It is important to note that traditional NSAIDs function as non-selective inhibitors of both COX − 1 and COX-2 enzymes. However, the presence of COX − 1 in the stomach, kidney, and platelets can result in an increased risk of adverse reactions, including gastrointestinal damage and bleeding (22). Selective COX-2 inhibitors, in contrast, specifically target COX-2 activity while exerting minimal influence on COX − 1; however, apprehensions have arisen regarding their cardiovascular safety profile and nephrotoxicity. Similarly, opioids employed for the management of moderate-to-severe pain are accompanied by notable adverse effects such as nausea, vomiting, constipation, respiratory depression, and cognitive impairment (23). These untoward effects have the potential to protract hospitalization duration, escalate hospitalization expenses, and induce anxiety among patients.

In the context of TKA surgery, there exist two primary categories: those in which the posterior cruciate ligament (PCL) is preserved and those in which it is not. A randomized controlled study (24) was conducted to investigate the effects of PCL preservation on patients who received a prosthesis. The hypothesis was that by maintaining the PCL, mechanical stress would stimulate PCL proprioceptors, leading to the activation of nerve reflexes and subsequent tightening of relevant muscles and ligaments. However, due to the loss of normal function in most patients, the formation of nerve reflexes proved challenging. Conversely, the prosthesis that replaces the posterior cruciate ligament does not maintain the integrity of the ligament itself. Instead, it utilizes a post and cassette structure to facilitate the rolling back mechanism and enhance knee joint flexion. Comparisons between TKA procedures with and without preservation of the PCL revealed no significant differences in VAS pain scores and HSS scores. The potential retention of proprioceptive sensation during knee flexion in patients with preserved PCL prostheses warrants further investigation to determine its potential benefits for subsequent knee rehabilitation (25). Danilo De Oliveira Silva’s findings (26) indicate that most of the patients (85%) who had undergone TKA using a posterior-stabilized (PS) prosthesis with routine patellar resurfacing reported absence of anterior knee pain at 12 months following surgery. The study conducted by White et al. (27) revealed that undergoing TKA, the Attune PS prosthesis exhibited a significantly lower overall occurrence of anterior knee pain (AKP) and knee crepitation in comparison with the PFC Sigma prosthesis (12.5% vs. 25.8%, *p* < 0.05). The occurrence of painful tremor was lower in both groups (1.0% vs. 4.1%), with no statistically significant difference, and there were no significant disparities observed in the four postoperative indices, namely, the HSS score, WOMAC score, ROM, and patient satisfaction. Both prostheses are extensively utilized in clinical practice, and the prevalence of residual knee pain was minimal after a 2-year follow-up period. A group of 47 TKA prostheses applied to 39 patients (32 women) were analyzed retrospectively. All the prostheses had been implanted by the same team of orthopedic surgeons using the same surgical method (cemented, with patella prosthesis and posterior stabilization with the sacrifice of the posterior cruciate ligament). It was found that male sex were considered good outcome predictors, underlying the importance of considering sex in understanding postoperative pain course; meanwhile, old preoperative age was considered as unfavorable underlying (28).

Due to the limitations of pharmacological therapy, a number of patients have turned to acupuncture as a supplementary and alternative treatment. Traditional Chinese Medicine (TCM) analgesia holds a crucial position in clinical practice for managing perioperative pain and should not be disregarded. Extensive research has consistently focused on acupuncture analgesia, with multiple clinical studies documenting its effectiveness in alleviating postoperative pain following TKA (29). Clinical reports on the utilization of acupuncture anesthesia in thyroid surgery and thoracic surgery have been documented as early as the 1980s. By integrating traditional Chinese medicine’s meridian and acupoint theory with nerve electrical stimulation technology, electroacupuncture and transcutaneous electrical acupoint stimulation present notable alternative therapeutic options (30). These methods not only exhibit clinical effectiveness but also mitigate labor costs and technical challenges associated with acupuncture treatment, while concurrently minimizing adverse reactions such as needle bending, bleeding, and infection. Research studies have demonstrated that the integration of transcutaneous electrical acupoint stimulation and electroacupuncture in the perioperative management of patients undergoing total knee arthroplasty (TKA) effectively mitigates pain, diminishes limb edema, expedites recovery (31), and aligns with the principles of ERAS. This incorporation of acupuncture techniques broadens the scope of clinical applications and garners acceptance from a growing cohort of surgeons. The ear is intricately linked to the body’s meridians, and auricular therapy represents a unique modality within the realm of acupuncture and moxibustion. This therapy involves the stimulation of specific areas on the auricle, known as auricular points, through the application of acupuncture or other methods, with the aim of diagnosing and treating various diseases (32). Numerous clinical studies have demonstrated the effectiveness of auricular point therapy in alleviating conditions such as traumatic pain (33), headache (34), postoperative wound pain, and neuropathic pain (35).

The study of the analgesic properties of acupuncture serves as a connection between traditional Chinese medicine and modern Western medicine. Through fundamental research, new insights have been gained regarding the mechanisms underlying acupuncture-induced pain relief, including the involvement of peripheral purine signaling (36) and TRPV channels (37), which expand upon the established central opioid peptide analgesia paradigm (38). Simultaneously, acupuncture therapies have been shown to modulate pain sensation, emotion, and memory (39), thereby enhancing the understanding of its analgesic mechanisms. The field of acupuncture research has long grappled with the challenge of bridging the gap between basic research and clinical practice, but there is growing anticipation that the analgesic mechanisms of acupuncture therapy will be increasingly validated through clinical trials. The four commonly utilized acupuncture-related therapies in clinical practice are traditional acupuncture, electroacupuncture, transcutaneous electrical acupoint stimulation, and auricular point therapy. Previous studies on postoperative pain following total knee arthroplasty have primarily compared various acupuncture-related therapies collectively against other interventions, emphasizing pairwise comparisons. Despite the widespread utilization of acupuncture, the existing evidence on its efficacy appears to be inconclusive. Various studies have yielded divergent findings. One systematic review demonstrated that acupuncture may improve postoperative pain management after total knee arthroplasty (TKA) (40), while a clinical trial suggested that acupuncture was not superior to pharmacological treatments (41). This study employed a network meta-analysis (NMA) approach to evaluate and rank four distinct types of acupuncture-related interventions in conjunction with multimodal analgesia for post-TKA pain relief. This study seeks to utilize the benefits of combining traditional Chinese and Western medicine to offer evidence-based recommendations for selecting the most optimal treatment approach in clinical settings.

## 2 Materials and methods

Our study was conducted based on the checklist of the preferred reporting items for systematic reviews and meta-analyses for network meta-analysis (PRISMA-NMA) guidelines (42) (Supplementary Appendix 1) and reporting items of systematic reviews and meta-analyses involving acupuncture (43). The PRISMA-NMA and PRISMA for acupuncture checklists are both updates to PRISMA. Based on the 27 items from the PRISMA checklist, the PRISMA-NMA checklist revised 11 items related to NMA and added 5 new items to guide and improve the writing and reporting for NMA, while PRISMA for acupuncture checklist revised 6 items related to acupuncture operations and added 5 new items to be better used for the systematic review about acupuncture therapies. The research has been registered in PROSPERO, with the registration website https://crd.york.ac.uk/PROSPERO/#recordDetails and registration number CRD42023492859.

### 2.1 Literature retrieval strategy

**Figure 1:**
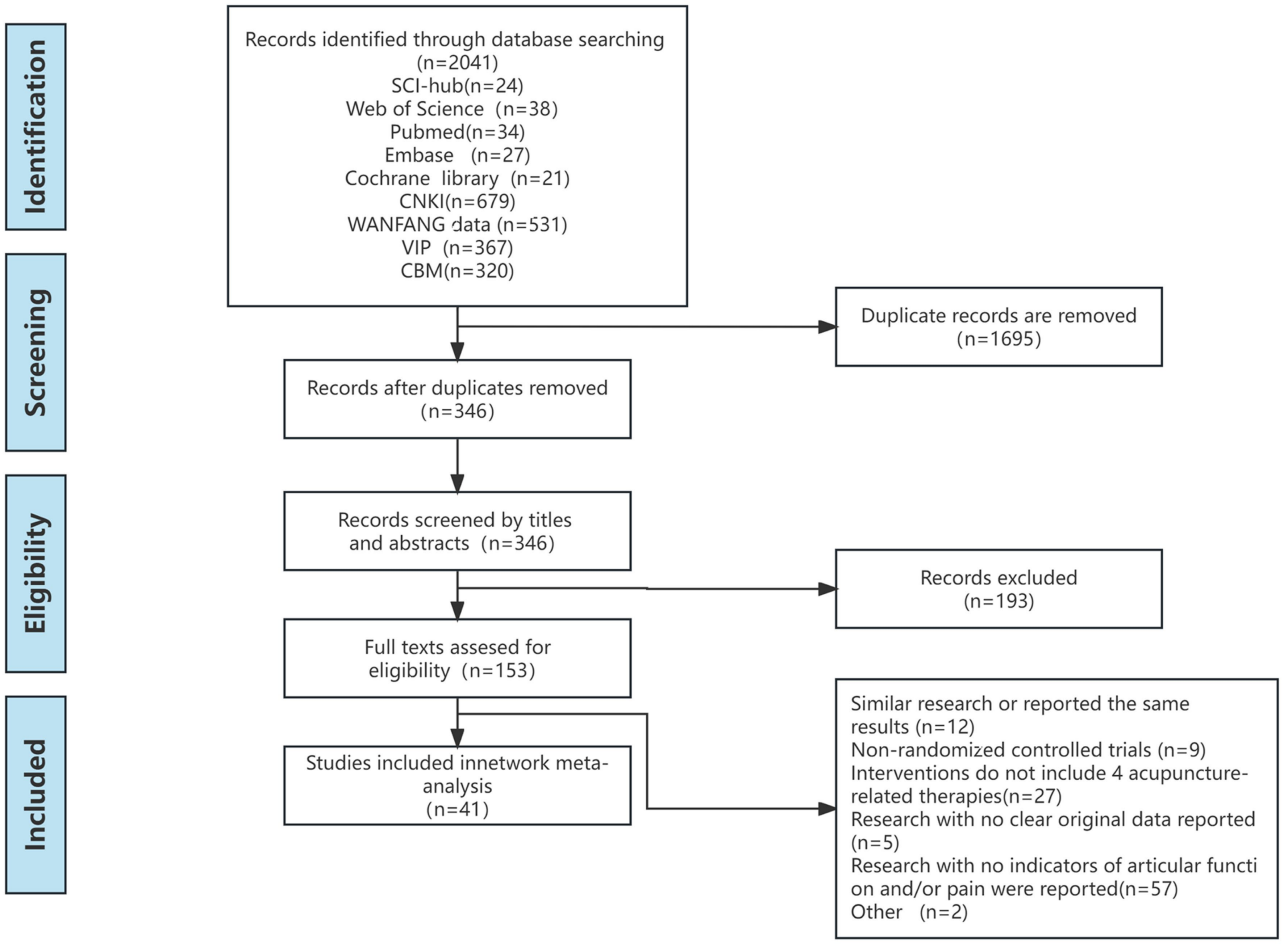
Study flow diagram.

A computer search was conducted in five English databases (Web of Science, PubMed, SCI-hub, Embase, and Cochrane Library) and four Chinese databases [China Biology Medicine (CBM), China National Knowledge Infrastructure (CNKI), Wanfang Data, and Chinese Scientific Journal Database (VIP)] to collect all randomized controlled trials (RCTs) on acupuncture therapy for pain after TKA from the establishment of the database until November 1, 2023 (Supplementary Appendix 2). The retrieval strategy employed a combination of subject headings and free words which were adjusted based on different retrieval systems. Retrieval terms included acupuncture, electroacupuncture, auricular acupuncture, transcutaneous electrical acupoint stimulation, total knee arthroplasty, multimodal analgesia, and pain (Figure 1).

### 2.2 The literature inclusion criteria

(1) The literature utilized in this study was a prospective randomized controlled trial (RCT). The inclusion criteria for the cases involved a diagnosis of knee osteoarthritis (44) based on the established diagnostic criteria for knee osteoarthritis (refer to Table 1). The cases were required to meet the first criterion and any two of the second, third, fourth, and fifth criteria. Additionally, the severity of knee osteoarthritis was assessed through the grading of knee X-ray films using the Kellgren-Lawrence method (45) (refer to Table 2). Subjects with Kellgren-Lawrence grades III and IV, necessitating TKA surgical intervention (46), will be encompassed. No limitations will be imposed on the patients’ age, gender, or race.
(2) The treatment group was administered various interventions, including acupuncture, transcutaneous electrical acupoint stimulation, electroacupuncture, and auricular acupuncture, in conjunction with multimodal analgesia (including one or more of analgesics, nervous block, and patient-controlled analgesia pump), while the control group solely received multimodal analgesia.
(3) The literature should contain original data pertaining to postoperative VAS score, preoperative and postoperative HSS score, preoperative and postoperative ROM measurements, preoperative and postoperative PPT score, and postoperative adverse events.
(4) The language of the literature could be either Chinese or English.

**Table 1:** Diagnostic criteria utilized for identifying osteoarthritis of the knee.

Serial number	Symptoms or signs
1	Recurrent knee pain in the past month
2	The X-ray film, taken in a standing or weight-bearing position, revealed the presence of joint space narrowing, subchondral sclerosis and/or cystic degeneration, as well as osteophytes at the articular edge
3	Individuals aged over 50 years
4	Morning stiffness duration of less than 30 min
5	Audible bone friction sound (or sensation) experienced during physical activity

**Table 2:** Kellgren-Lawrence grading system for osteoarthritis.

Grade	Radiologic findings of knee osteoarthritis
0	No radiological findings of knee osteoarthritis
I	Doubtful narrowing of joint space and possible osteophytic lipping
II	Definite osteophytes and possible narrowing of joint space
III	Moderate multiple osteophytes, definite narrowing of joint space, small pseudocystic areas with sclerotic walls and possible deformity of bone contour
IV	Large osteophytes, marked narrowing of joint space, severe IV sclerosis, and definite deformity of bone contour

### 2.3 Exclusion criteria for literature

(1) The subjects in this study were diagnosed with knee arthritis, as indicated in Table 1. However, their Kellgren-Lawrence classification ranged from 0 to II, as shown in Table 2. Consequently, non-surgical therapy was chosen over TKA. The objective of this research was to conduct a randomized controlled trial to evaluate the efficacy of various acupuncture therapies in alleviating pain associated with knee arthritis.
(2) Non-randomized studies (NRSs) include non-randomized controlled clinical trials, self-control studies, historical control studies, cohort studies, case–control studies, and cross-sectional survey studies.
(3) Other non-penetrating stimuli (such as point ion penetration, laser irradiation, and magnetic therapy), other penetrating stimuli (such as head acupuncture, point injection, and point embedding), and special acupuncture methods (such as triangle needle, fire needle, and acupotomy).
(4) The control group received additional interventions, including psychological counseling and massage therapy.
(5) There was no original data in the literature, and the author could not be contacted.
(6) The authors of the literature were the same or showed the similar data results.

### 2.4 Primary and secondary outcomes

#### 2.4.1 Primary outcome indicators

The primary outcome measures encompass two dimensions: first, the pain intensity experienced by the participants following TKA; second, the functional status of the knee joint in the participants prior to and post-TKA.

(1) Visual analog scale (VAS) score (47): A 10 cm long straight line is drawn on a blank sheet of paper, with “no pain” and “most severe pain” labeled at each end. The patient is instructed to indicate their pain level and psychological perception by marking a point on the line. The distance from the starting point to the marked point represents the intensity of pain. The study aims to assess the disparities in the VAS score between the third and seventh day after surgery, as well as the baseline value.
(2) The pain pressure threshold (PPT) (48) will be measured by applying pressure to the medial side of the knee joint, within a 3 cm range from the midpoint of the medial edge of the patella, using a digital pressure tester. The pressure will be gradually increased until the patient experiences pain, at which point it will be immediately ceased and the corresponding value recorded. The discrepancy between the PPT and the baseline value will then be calculated.
(3) The evaluation of the knee joint’s performance at hospital for special surgery (HSS) (49) encompasses various aspects, including pain, function, activity, muscle strength, flexion deformity, stability, and other relevant factors. A higher score on the HSS scale indicates better knee function. Additionally, the disparity between the HSS score and the initial value is computed to determine the improvement.
(4) The range of motion (ROM) (49) of the knee joint is assessed by measuring the extent of movement before and after TKA using a joint ruler. The discrepancy between ROM and the baseline value is then calculated.

#### 2.4.2 Secondary outcome indicators

To further assess the effectiveness and safety of various acupuncture therapies, secondary outcome measures including the occurrence of postoperative nausea and vomiting, dizziness, and drowsiness were employed.

### 2.5 Literature screening and data extraction

Two researchers (YH and YX) independently conducted a comprehensive literature review and meticulously extracted relevant data. In concrete terms, the title and abstract were read and checked to exclude literature that did not meet the inclusion criteria. Then, the full texts of the potentially eligible studies were read to identify studies that could be included in this quantitative analysis. The results were cross-checked for accuracy, and in the event of any disagreement, a third researcher was consulted to reach a consensus.

Regarding data extraction, Excel 2016 was utilized to construct a systematic table encompassing pertinent information from eligible studies. This included: (1) general information: first author’s name, journal title, publication date, grouping details, and sample sizes in each group; (2) baseline characteristics: age distribution, disease duration, and pre-treatment measurements for each outcome indicator within every group; (3) intervention measures: type of acupuncture therapy administered, treatment frequency, and duration of treatment cycles; and (4) outcome indicators’ data along with post-treatment adverse reactions.

### 2.6 Assessment of risk bias in included studies

The Cochrane Handbook 5.1 was utilized for the assessment of risk bias in the included studies, encompassing randomization, assignment concealment, blinding of patients and staff, blinding of outcome assessment, incomplete outcome data, selective outcome reporting, and other potential sources of bias (50, 51). In RevMan 5.4 software, we categorized the levels of risk as “high,” “low,” or “unclear,” respectively.

### 2.7 Statistical analysis

Software programs RevMan (Version 5.4) and Stata (Version16.0) were used to do statistical analysis. The study utilized odds ratios (ORs) and 95% confidence intervals (95% CIs) for dichotomous variables and mean differences ± standardized mean differences (SMD) and 95% CI for continuous variables.

Bias risk assessment was conducted using RevMan 5.4 software, and frequency science network meta-analysis was performed using Stata16.0. Data processing, network evidence graph drawing, network league table creation, and calculation of the surface under the cumulative ranking curve (SUCRA) were performed using the network and mvmeta package commands. The study was divided and restructured into all-paired two-arm trials if it had been a three-arm trial (52). In instances where closed loops were observed in the network evidence graph, indicating discrepancies among research findings, the ifplot command was employed to identify inconsistencies. The inconsistency factor (IF) value and its corresponding 95% confidence interval were computed for each closed loop to evaluate the consistency of direct and indirect comparison results. The fitting inconsistency test was performed on the closed loops formed in the network meta-analysis. If *p* > 0.05, the inconsistencies in direct and indirect comparisons were not statistically significant, and a consistency model could be used for meta-analysis. If there was no closed loop, the consistency model could be used directly. The SUCRA was generated with Stata16.0, which shows the SUCRA scores for all interventions, with higher SUCRA values denoting a higher treatment class. The closer the value was to 100%, the better the efficacy of the intervention (53). A funnel plot was constructed using Stata16.0 to examine potential publication bias within the included studies (54).

## 3 Results

### 3.1 Literature retrieval

After primary retrieval, a total of 2041 relevant literature were identified, comprising 1897 Chinese and 144 English publications. Following the removal of 1,695 duplicate articles, the remaining literature underwent screening based on titles and abstracts, resulting in the exclusion of 193 studies. Subsequently, after full-text evaluation, an additional 112 literature were excluded. Finally, a total of 41 literature (55–94) were included in this review (Figure 1 illustrates the specific inclusion process).

### 3.2 Basic characteristics of the included studies

In these selected studies (*n* = 41), four types of acupuncture therapy interventions were investigated: acupuncture alone, electroacupuncture, transcutaneous electrical acupoint stimulation (TEAS), and auricular therapy. These interventions were combined with perioperative multimodal analgesia. Among them, one study employed a three-arm trial design (57), while the rest utilized two-arm trials. Tables 3, 4 present detailed information regarding baseline characteristics and acupuncture methods used in the included studies.

**Table 3:** Baseline characteristics of the included studies.

References	Country	Intervention			Control			Course	Type of outcomes	Adverse reaction
		Treatment	n	Years (^−^χ ± s)	Treatment	n	Years (^−^χ ± s)			
Yang et al.	China	EA + MA	30	67.30 ± 0.00	MA	30	68.50 ± 0.00	1 W	VAS, ROM	NR
Chen et al.	China	EA + MA	20	67.10 ± 7.00	MA	20	66.70 ± 6.30	1 W	VAS	NR
Kang et al.	China	EA + MA	63	70.19 ± 5.42	MA	63	70.46 ± 4.90	1 W	PPT	NR
Zhu	China	EA + MA	16	69.29 ± 6.43	MA	16	66.56 ± 6.69	2 W	VAS, ROM	NR
Ling et al.	China	ACU + MA	30	65.73 ± 7.29	MA	30	64.27 ± 6.13	1 W	VAS, HSS	NR
Zheng et al.	China	ACU + MA	30	67.80 ± 8.12	MA	30	70.70 ± 8.47	1 W	VAS	NR
Zhen et al.	China	TEAS+MA	25	65.92 ± 7.60	MA	25	65.64 ± 7.15	1 W	VAS, HSS	Reported
Wei et al.	China	TEAS+MA	49	66.26 ± 7.35	MA	49	67.90 ± 7.69	2 W	VAS, HSS	Reported
Xiang et al.	China	EA + MA	41	63.00 ± 5.00	MA	61	63.00 ± 6.00	2 W	VAS, ROM	Reported
Zhang	China	ACU + MA	43	63.12 ± 8.31	MA	43	62.75 ± 8.19	2 W	VAS, HSS	NR
Ma et al.	China	EA + MA	41	69.63 ± 7.60	MA	41	70.92 ± 6.55	5D	VAS, HSS	NR
Zhang et al.	China	ACU + MA	30	67.00 ± 7.00	MA	30	67.00 ± 9.00	2 W	VAS, ROM, HSS	Reported
Zhu et al.	China	ACU + MA	45	49.23 ± 5.61	MA	45	48.23 ± 5.41	1 W	VAS	Reported
Liao et al.	China	ACU + MA	30	70.00 ± 4.67	MA	30	71.37 ± 3.15	2 W	VAS	NR
Liu et al.	China	ACU + MA	20	65.26 ± 7.23	MA	20	67.32 ± 6.54	1 W	VAS	Reported
Li et al.	China	ACU + MA	20	67.90 ± 7.50	MA	20	66.8 ± 6.3	2 W	VAS, ROM	NR
Wang	China	ACU + MA	54	53.29 ± 4.52	MA	51	54.13 ± 6.24	2 W	VAS, HSS, ROM	NR
Wu et al.	China	TEAS+MA	60	70.75 ± 8.23	MA	60	70.55 ± 7.44	4 W	VAS, HSS	NR
Zhang et al.	China	TEAS+MA	40	69.20 ± 3.00	MA	40	68.60 ± 3.20	3D	VAS	NR
Chen et al.	China	TEAS+MA	30	63.37 ± 5.81	MA	30	65.17 ± 5.78	1 W	VAS	Reported
Bai et al.	China	TEAS+MA	71	65.61 ± 5.73	MA	69	65.07 ± 5.98	1 W	VAS, HSS	Reported
Xu et al.	China	TEAS+MA	46	67.00 ± 7.00	MA	45	67.70 ± 7.30	2 W	VAS, ROM	NR
Wang et al.	China	TEAS+MA	45	70.00 ± 4.00	MA	45	71.00 ± 6.00	3D	VAS	Reported
Tsang et al.	Hong Kong, China	ACU + MA	12	70.60 ± 5.80	MA	12	66.10 ± 7.50	1 W	VAS, ROM	NR
Tzeng et al.	Taiwan, China	EA + MA	16	69.60 ± 5.60	MA	17	70.10 ± 6.90	2D	VAS	Reported
		no-point group	14	71.40 ± 7.30						
Chen et al.	China	EA + MA	30	68.90 ± 9.00	MA	30	69.00 ± 8.60	1 W	VAS	Reported
Lan	China	EA + MA	20	61.72 ± 9.94	MA	20	64.45 ± 8.43	1 W	VAS + ROM	Reported
Sheng	China	EA + MA	20	67.05 ± 7.04	MA	20	66.70 ± 6.28	1 W	VAS + ROM	Reported
Guo	China	EA + MA	25	64.72 ± 7.28	MA	25	62.64 ± 6.16	1 W	VAS + ROM+HSS	Reported
Chen et al.	China	EA + MA	35	64.70 ± 5.10	MA	35	65.60 ± 5.10	2 W	ROM+HSS	NR
Zhong	China	EA + MA	55	69.33 ± 5.66	MA	55	68.42 ± 5.35	3D	HSS	NR
Tong et al.	China	AAT + MA	30	70.00 ± 4.79	MA	30	71.37 ± 3.18	1 W	VAS + ROM+HSS	Reported
Yan	China	AAT + MA	30	70.00 ± 4.78	MA	30	71.37 ± 3.18	3D	VAS	NR
Zhang	China	AAT + MA	23	62.00 ± 8.00	MA	23	61.00 ± 9.00	2D	VAS	Reported
Kong et al.	China	AAT + MA	40	59.16 ± 6.42	MA	40	61.28 ± 5.63	1 W	VAS + ROM	NR
Du et al.	China	AAT + MA	30	65.02 ± 3.00	MA	30	64.38 ± 2.77	3D	VAS	Reported
Yan et al.	China	AAT + MA	102	62.20 ± 9.10	MA	102	64.10 ± 8.20	1 W	VAS + HSS	NR
Zhong et al.	China	AAT + MA	50	65.75 ± 1.41	MA	50	64.60 ± 0.70	3D	VAS + HSS	NR
Fu	China	AAT + MA	30	64.6 ± 7.29	MA	30	66.57 ± 6.96	3D	VAS + HSS	Reported
Wang et al.	China	AAT + MA	31	60.19 ± 6.33	MA	29	66.02 ± 7.76	3D	VAS	Reported
He	China	AAT + MA	45	62.56 ± 6.10	MA	45	61.58 ± 6.66	1 W	VAS + ROM+HSS	Reported

ACU, acupuncture; EA, electroacupuncture; TEAS, transcutaneous electrical acupoint stimulation; AAT, auricular acupoint therapy; MA, multimodal analgesia; NR, No reported; W, week; D, day; VAS, visual analog scale; ROM, the range of motion; HSS, hospital for special surgery; PPT, pain pressure threshold.

**Table 4:** Descriptions of the included acupuncture and related therapies.

References	Study of acupuncture	Names of acupuncture points used	Retention time	Acupuncturist qualifications	Acupuncture reaction	Frequency and course of acupuncture
Yang et al.	EA	Qimen (SP11), Sanyinjiao (SP06), Biguan (ST31), Fenglong (ST40)	30 min	Reported	Deqi	Once a day for 1 week
Chen et al.	EA	Liangqiu (ST34), Xuehai (SP10), Yinlingquan (SP09), Zusanli (ST36), Fenglong (ST40), Qiuxu (GB40)	30 min	NR	Deqi	Once a day for 1 week
Kang et al.	EA	Futu (ST32), Zusanli (ST36), Yinlingquan (SP09), Yanglingquan (GB34)	20 min	NR	Deqi	Once a day, 5 times for 1 week
Zhu	EA	Liangqiu (ST34), Xuehai (SP10), Yinlingquan (SP09), Zusanli (ST36), Fenglong (ST40), Qiuxu (GB40)	30 min	Reported	NR	NR
Ling et al.	ACU	Jianzhong (GB21), Linggu (GV20), Taibai (SP03)	NR	NR	Deqi	Once a day for 1 week
Zheng et al.	ACU	Xuehai (SP10), Liangqiu (ST34), Zusanli (ST36), Yanglingquan (GB34), Sanyinjiao (SP06), Taixi (KI03), Taichong (LR03), Hegu (LI04)	30 min	Reported	Deqi	Once a day for 1 week
Zhen et al.	TEAS	Hegu (LI04), Neiguan (PC06), Xuehai (SP10), Liangqiu (ST34), Yinlingquan (SP09), Zusanli (ST36)	30 min	NR	Deqi	Once a day for 1 week
Wei et al.	TEAS	Zusanli (ST36), Xuehai (SP10), Liangqiu (ST34), Yinlingquan (SP09), Taichong (LR03), Weizhong (BL40)	30 min	Reported	Deqi	3 times 1 day for 2 weeks
Xiang et al.	EA	Zusanli (ST36), Yinlingquan (SP09), Yanglingquan (GB34)	30 min	Reported	Deqi	Once a day for 1 week
Zhang	ACU	Xuehai (SP10), Yinmen (BL37), Yinlingquan (SP09), Yanglingquan (GB34), Chengfu (BL36)	NR	Reported	Deqi	Once a day for 2 weeks
Ma et al.	EA	Shousanli (LI10), Quchi (LI11), Zhouliao (LI12), Binao (LI14), Chize (LU05), Sidu (SJ09)	20 min	Reported	Deqi	Once a day for 5 days
Zhang et al.	ACU	Taichong (LR03), Kunlun (BL60), Shousanli (LI10), Quchi (LI11), Chize (LU05), Houxi (SI03), Shenmai (BL62), Sanyinjiao (SP06)	30 min	NR	Deqi	Once a day for 2 weeks
Zhu et al.	ACU	Chengfu (BL36), Taichong (LR03), Yinlingquan (SP09)	NR	NR	Deqi	Once a day for 1 week
Liao et al.	ACU	Taichong (LR03), Hegu (LI04)	30 min	NR	Deqi	Once a day for 2 weeks
Liu et al.	ACU	Taichong (LR03), Hegu (LI04), Liangqiu (ST34), Zhongdu (LR06)	30 min	NR	Deqi	Once a day for 3 days
Li et al.	ACU	Taichong (LR03), Kunlun (BL60), Yanglingquan (GB34), Zusanli (ST36), Hegu (LI04), Sanyinjiao (SP06)	30 min	Reported	Deqi	Once a day for 2 weeks
Wang	ACU	Chengfu (BL56), Yinmen (BL37), Xuehai (SP10), Yinlingquan (SP09), Yanglingquan (GB34), Taichong (LR03)	NR	NR	Deqi	Once a day for 2 weeks
Wu et al.	TEAS	Liangqiu (ST34), Zusanli (ST36), Yinlingquan (SP09), Yanglingquan (GB34), Xuehai (SP10), Yinshi (ST33)	30 min	NR	Deqi	Once a day for 4 weeks
Zhang et al.	TEAS	Hegu (LI04), Neiguan (PC06)	30 min	NR	Deqi	Once a day for 3 days
Chen et al.	TEAS	Zusanli (ST36), Xuehai (SP10), Liangqiu (ST34), Yinlingquan (SP09), Yanglingquan, (GB34), Weizhong (BL40), Hegu (LI04), Neiguan (PC06)	30 min	NR	Deqi	3 times 1 day for 1 week
Bai et al.	TEAS	Zusanli (ST36), Weizhong (BL40), Yanglingquan (GB34), Xuehai (SP10), Liangqiu (ST34), Yinlingquan (SP09)	30 min	NR	Deqi	3 times 1 day for 1 week
Xu et al.	TEAS	Zusanli (ST36), Weizhong (BL40), Xuehai (SP10), Liangqiu (ST34), Yinlingquan (SP09), Yanglingquan (GB34)	30 min	Reported	Deqi	Twice a day for 1 week
Wang et al.	TEAS	Hegu (LI04), Neiguan (PC06), Zusanli (ST36)	30 min	NR	Deqi	NR
Tsang et al.	ACU	Futu (ST32), Yinshi (ST33), Fengshi (GB31), Yangjiao (GB35), Yanglingquan (GB34), Zusanli (ST36)	20 min	Reported	Deqi	NR
Tzeng et al.	EA	Zusanli (ST36), Yanglingiquan (GB34)	30 min	Reported	Deqi	Once a day for 2 days
Chen et al.	EA	Zusanli (ST36), Xuehai (SP10), Liangqiu (ST34), Weizhong (BL40), Xiguan (LR07)	30 min	Reported	Deqi	Before the start of surgery
Lan	AAT	Xuehai (SP10), Liangqiu (ST34), Yinlingquan (SP09), Yanglingquan (GB34)	30 min	NR	Deqi	Once a day for 1 week
Sheng	AAT	Liangqiu (ST34), Xuehai (SP10), Yinlingquan (SP09), Zusanli (ST36), Fenglong (ST40), Qiuxu (GB40)	30 min	Reported	Deqi	Once a day for 1 week
Guo	AAT	Hegu (LI04), Zusanli (ST36), Taichong (LR03), Taixi (KI03), Yinlingquan (SP09), Yanglingquan (GB34)	30 min	Reported	Deqi	Once a day for 1 week
Chen et al.	AAT	Xuehai (SP10), Liangqiu (ST34), Dubi (ST35), Neixiyan (EX-LE4), Yanglingquan (GB34), Zusanli (ST36), Kunlun (BL60)	30 min	NR	Deqi	Once a day for 2 weeks
Zhong	AAT	Futu (ST32), Zusanli (ST36), Yanglingquan (GB34), Yinlingquan (SP09)	20 min	Reported	Deqi	Once a day for 3 days
Tong et al.	AAT	Shenmen (TF4), Subcortex (AT4), Sympathetic (AT1), Knee (TG1)	5 min	NR	Deqi	Once every 30 min for 1 week
Yan	AAT	Shenmen (TF4), Knee (TG1), Kidney (TG2), Small occipital nerve point, Large auricular nerve point	5 min	NR	Deqi	Once every 2 h for 3 days
Zhang	AAT	Shenmen (TF4), Subcortex (AT4), Knee (TG1)	10 min	NR	Deqi	3 to 5 times 1 day for 3 days
Kong et al.	AAT	Shenmen (TF4), Subcortex (AT4), Sympathetic (AT1), Endocrine (AT2), Knee (TG1)	3 min	Reported	Deqi	Twice 1 day for 1 week
Du et al.	AAT	Shenmen (TF4), Subcortex (AT4), Sympathetic (AT1), Knee (TG1), Kidney (TG2), Spleen (AT5), Stomach (AT6)	2 min	Reported	Deqi	4 times 1 day for 3 days
Yan et al.	AAT	Shenmen (TF4), Subcortex (AT4), Sympathetic (AT1), Knee (TG1)	5 min	Reported	Deqi	3 times 1 day for 2 week
Zhong et al.	AAT	Shenmen (TF4), Subcortex (AT4), Knee (TG1), Kidney (TG2)	3 min	Reported	Deqi	4 times 1 day for 3 days
Fu	AAT	Shenmen (TF4), Subcortex (AT4), Knee (TG1), Kidney (TG2)	2 min	Reported	Deqi	4 times 1 day for 3 days
Wang et al.	AAT	Shenmen (TF4), Subcortex (AT4), Knee (TG1), Lung (AT3)	5 min	Reported	Deqi	Operation when feeling the pain
He	AAT	Shenmen (TF4), Subcortex (AT4), Sympathetic (AT1), Knee (TG1)	3 min	Reported	Deqi	4 times 1 day for 1 week

ACU, acupuncture; EA, electroacupuncture; TEAS, transcutaneous electrical acupoint stimulation; AAT, auricular acupoint therapy; MA, multimodal analgesia; NR, no reported.

### 3.3 Quality evaluation of the included literature

**Figure 2:**
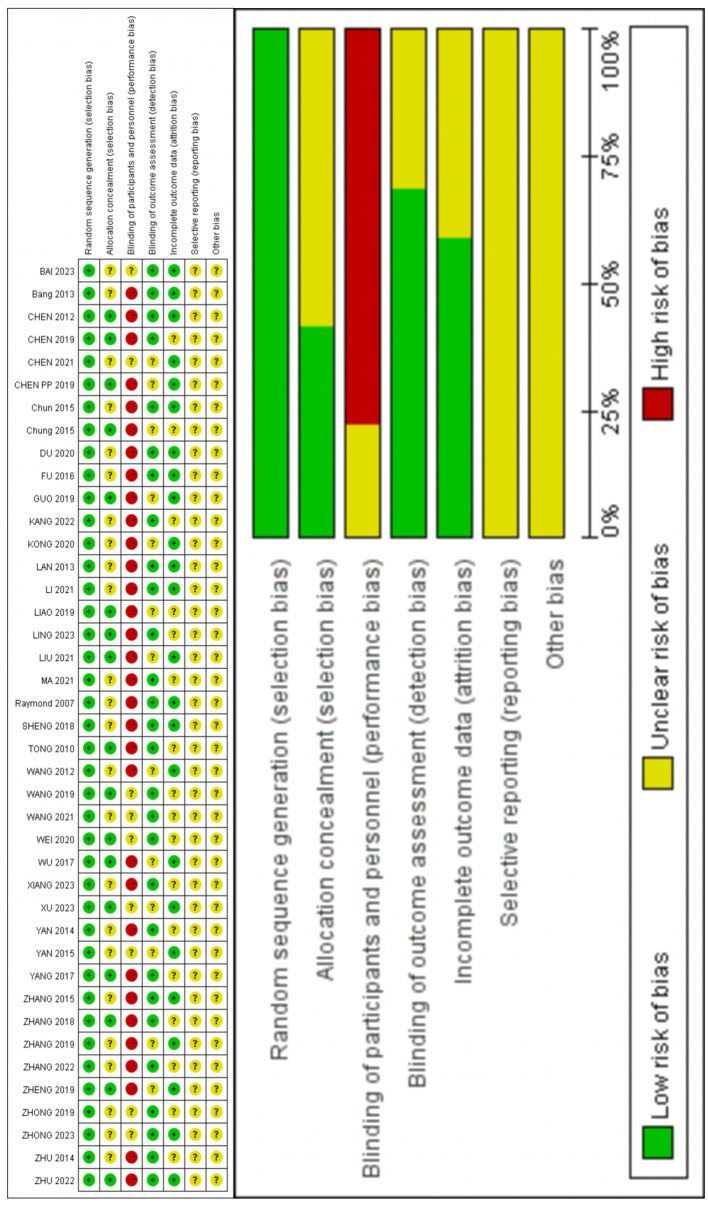
Quality assessment of included studies.

The risk bias assessment was independently conducted by two researchers (DZ and LW). The evaluation criteria consisted of seven items including randomization method, allocation concealment method, and blinding procedures for participants or outcome assessment. Of all the trials reviewed (*n* = 41), 41 (100%) described their random sequence generation process, while only 17 (42%) reported their allocation concealment method. None of the studies implemented blinding techniques for participants or outcome evaluation. Twenty-seven trials (65%) provided complete outcome data. It should be noted that due to the nature of acupuncture treatment involving active participation from both practitioners and patients during administration, implementing blind methods is challenging. The results of risk assessment are shown in Figure 2.

The quality of evidence of the included studies was evaluated according to the GRADE assessment method, and seven outcome indicators were analyzed. Evidence for VAS-3, HSS, ROM, incidence of PODS, and incidence of PONV was of medium quality. The quality of evidence for VAS-7 and PPT was low. The decline in quality was mainly caused by the limitations of randomization method and the blinding method as well as the imprecision due to the small sample size. The detailed quality assessment is shown in Table 5.

**Table 5:** GRADE assessment of outcome indicators.

Outcome indicator	Downgrading factors					Quality grade
	Boundedness	Inconsistency	Not directly	Inaccuracy	Publication bias	
VAS-3	−1	0	0	0	0	Middle rank
VAS-7	−1	0	0	0	−1	Low rank
PPT	−1	0	0	0	−1	Low rank
HSS	−1	0	0	0	0	Middle rank
ROM	−1	0	0	0	0	Middle rank
Incidence of PONV	−1	0	0	0	0	Middle rank
Incidence of PODS	−1	0	0	0	0	Middle rank

VAS, visual analog scale;VAS-3, VAS score assessed on the third postoperative day; VAS-7, VAS score assessed on the seventh postoperative day; ROM, the range of motion; HSS, hospital for special surgery; PPT, pain pressure threshold; incidence of PONV, incidence of postoperative nausea and vomiting; incidence of PODS, incidence of postoperative dizziness and sleepiness.

### 3.4 Results of network meta-analysis

#### 3.4.1 Evidence network diagram of intervention measures

**Figure 3:**
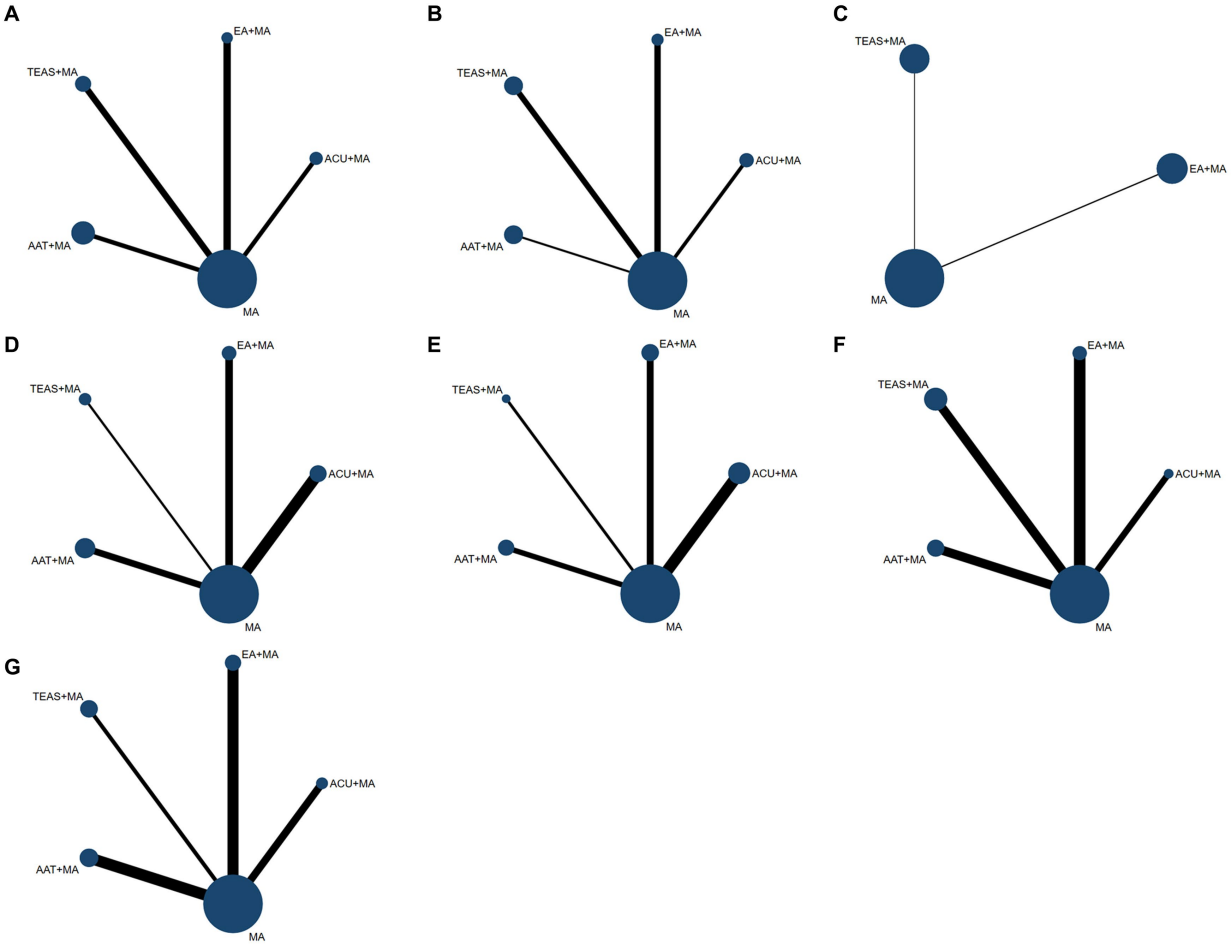
Network structure for treatment comparisons. **(A)** VAS-3 scores; **(B)** VAS-7 scores; **(C)** PPT scores; **(D)** HSS scores; **(E)** ROM scores; **(F)** incidence of postoperative nausea and vomiting; **(G)** incidence of postoperative dizziness and sleepiness. ACU, acupuncture; EA, electroacupuncture; TEAS, transcutaneous electrical acupoint stimulation; AAT, auricular acupoint therapy: MA, multimodal analgesia.

Stata16.0 was utilized to analyze the evidence network diagram of five intervention measures, where the size of each circle represents the sample size and the thickness of connecting lines indicates the number of RCTs using two intervention measures (Figure 3).

#### 3.4.2 Conflict detection

The evidence network diagram presented in Figure 3 indicated the absence of closed-loop formation for both primary and secondary outcome indicators, thereby precluding the need for an inconsistency test.

#### 3.4.3 Primary outcome indexes VAS-3, VAS-7, PPT, HSS, and ROM scores

(1) As shown in Figure 3A, a total of 29 RCTs mentioned VAS score on the third day after TKA, involving 2039 patients and 5 interventions including ACU + MA, EA + MA, TEAS+MA, AAT + MA, and MA. There was no significant difference in the efficacy of the five interventions in improving VAS score on the third day after TKA (Table 6).
(2) As shown in Figure 3B, a total of 21 RCTs mentioned VAS score on the seventh day after TKA, involving 1,447 patients and 5 interventions including ACU + MA, EA + MA, TEAS+MA, AAT + MA, and MA. In improving VAS score on the seventh day after TKA, TEAS+MA (SMD: 0.67; 95% CI: 0.01, 1.32) was superior to MA (Table 6).
(3) As shown in Figure 3C, a total of four RCTs mentioned postoperative PPT scores, involving 408 patients and 3 interventions: EA + MA, TEAS+MA, and MA. In terms of improving PPT scores after TKA, there was no significant difference in the efficacy of EA + MA, TEAS+MA, and MA interventions (Table 6).
(4) As shown in Figure 3D, a total of 15 RCTs mentioned postoperative HSS scores, involving 1,397 patients and 5 interventions including ACU + MA, EA + MA, TEAS+MA, AAT + MA, and MA. In terms of improving postoperative HSS scores after TKA, the three interventions including ACU + MA (SMD: 6.45; 95%CI: 3.30, 9.60), EA + MA (SMD: 4.89; 95%CI: 1.46, 8.32), and TEAS+MA (SMD: 5.31; 95%CI: 0.85, 9.78) were more effective than MA, which helped to promote early knee joint function recovery after TKA (Table 6).
(5) As shown in Figure 3E, a total of 18 RCTs mentioned postoperative ROM, involving 1,150 patients and 5 interventions including ACU + MA, EA + MA, TEAS+MA, AAT + MA, and MA. In terms of improving postoperative ROM scores after TKA, the efficacy of ACU + MA (SMD:7.43; 95%CI: 2.51, 12.36) was better than EA + MA, the efficacy of ACU + MA (SMD:10.12; 95%CI: 2.79, 17.45) was better than TEAS+MA, the efficacy of ACU + MA (SMD: 7.82; 95%CI: 2.21, 13.43) was better than AAT + MA, and the efficacy of ACU + MA (SMD: 9.74; 95%CI: 6.12, 13.37) was better than MA. It can be seen that ACU + MA has a significant advantage in improving postoperative ROM compared with other interventions (Table 6).

**Table 6:** Network meta-analysis results.

(A) VAS-3				
ACU + MA				
−0.33 (−1.21, 0.55)	EA + MA			
−0.33 (−1.25, 0.58)	−0.00 (−0.93, 0.93)	TEAS+MA		
0.28 (−0.53, 1.09)	0.62 (−0.21, 1.44)	0.62 (−0.25, 1.49)	AAT + MA	
−0.10 (−0.71, 0.51)	0.23 (−0.40, 0.86)	0.23 (−0.45, 0.92)	−0.38 (−0.92, 0.15)	MA
(B) VAS-7				
ACU + MA				
−0.46 (−1.27, 0.36)	EA + MA			
−0.80 (−1.66, 0.06)	−0.35 (−1.23, 0.54)	TEAS+MA		
0.01 (−0.87, 0.89)	0.47 (−0.44, 1.37)	0.81 (−0.13, 1.76)	AAT + MA	
−0.14 (−0.70, 0.42)	0.32 (−0.27, 0.91)	0.67 (0.01, 1.32)*	−0.15 (−0.83, 0.54)	MA
(C) PPT				
EA + MA				
−0.05 (−0.82, 0.73)	TEAS+MA			
0.37 (−0.20, 0.93)	0.41 (−0.12, 0.95)	MA		
(D) HSS				
ACU + MA				
1.56 (−3.08, 6.20)	EA + MA			
1.13 (−4.33, 6.60)	−0.42 (−6.05, 5.21)	TEAS+MA		
3.23 (−1.35, 7.81)	1.67 (−3.11, 6.46)	2.10 (−3.48, 7.67)	AAT + MA	
6.45 (3.30, 9.60)*	4.89 (1.46, 8.32)*	5.31 (0.85, 9.78)*	3.22 (−0.13, 6.56)	MA
(E) ROM				
ACU + MA				
7.43 (2.51, 12.36)*	EA + MA			
10.12 (2.79, 17.45)*	2.69 (−4.51, 9.89)	TEAS+MA		
7.82 (2.21, 13.43)*	0.39 (−5.05, 5.83)	−2.30 (−9.98, 5.38)	AAT + MA	
9.74 (6.12, 13.37)*	2.31 (−1.03, 5.65)	−0.38 (−6.75, 6.00)	1.92 (−2.37, 6.21)	MA
(F) Incidence of postoperative nausea and vomiting				
ACU + MA				
0.68 (0.20, 2.35)	EA + MA			
0.98 (0.30, 3.20)	1.45 (0.60, 3.47)	TEAS+MA		
1.47 (0.45, 4.82)	2.17 (0.89, 5.29)	1.50 (0.67, 3.36)	AAT + MA	
0.30 (0.11, 0.85)*	0.44 (0.22, 0.87)*	0.30 (0.17, 0.53)*	0.20 (0.11, 0.36)*	MA
(G) Incidence of postoperative dizziness and sleepiness				
ACU + MA				
1.84 (0.38, 8.85)	EA + MA			
2.81 (0.65, 12.26)	1.52 (0.51, 4.55)	TEAS+MA		
4.98 (1.01, 24.42)*	2.70 (0.77, 9.40)	1.77 (0.57, 5.45)	AAT + MA	
1.01 (0.27, 3.74)	0.55 (0.23, 1.30)	0.36 (0.18, 0.70)*	0.20 (0.08, 0.50)*	MA

ACU, acupuncture; EA, electroacupuncture; TEAS, transcutaneous electrical acupoint stimulation; AAT, auricular acupoint therapy; MA, multimodal analgesia; VAS, visual analog scale;VAS-3, VAS score assessed on the third postoperative day; VAS-7, VAS score assessed on the seventh postoperative day; ROM, the range of motion; HSS, hospital for special surgery; PPT, pain pressure threshold; incidence of PONV, incidence of postoperative nausea and vomiting; incidence of PODS, incidence of postoperative dizziness and sleepiness.*Represents positive results.

#### 3.4.4 Secondary outcome indicators: incidence of adverse reactions

(1) As shown in Figure 3F, a total of 21 RCTs mentioned the incidence of postoperative nausea and vomiting, involving 1,389 patients and 5 interventions: ACU + MA, EA + MA, TEAS+MA, AAT + MA, and MA. The occurrence of postoperative nausea and vomiting was significantly lower with ACU + MA (OR = 0.30; 95%CI: 0.11, 0.85), EA + MA (OR = 0.44; 95%CI: 0.22, 0.87), TEAS+MA (OR = 0.30; 95%CI: 0.17, 0.53), and AAT + MA (OR = 0.20; 95%CI: 0.11, 0.36) compared to MA (Table 6).
(2) As shown in Figure 3G, a total of 16 RCTs mentioned the incidence of postoperative dizziness and drowsiness, involving 1,076 patients and 5 interventions: ACU + MA, EA + MA, TEAS+MA, AAT + MA, and MA. The occurrence of postoperative dizziness and drowsiness was higher with ACU + MA (OR = 4.98; 95%CI: 1.01, 24.42) than with AAT + MA; the incidence of postoperative dizziness and drowsiness was lower with TEAS+MA (OR = 0.36; 95%CI: 0.18, 0.70) and AAT + MA (OR = 0.20; 95%CI: 0.08, 0.50) than with MA (Table 6).

The incidence of adverse reactions in the study utilizing acupuncture-related therapy as an intervention measure was generally lower compared to that of the simple multimodal analgesia scheme, thereby indicating the safety and efficacy of applying acupuncture-related therapy during the perioperative period of TKA.

#### 3.4.5 SUCRA probability ranking

**Figure 4:**
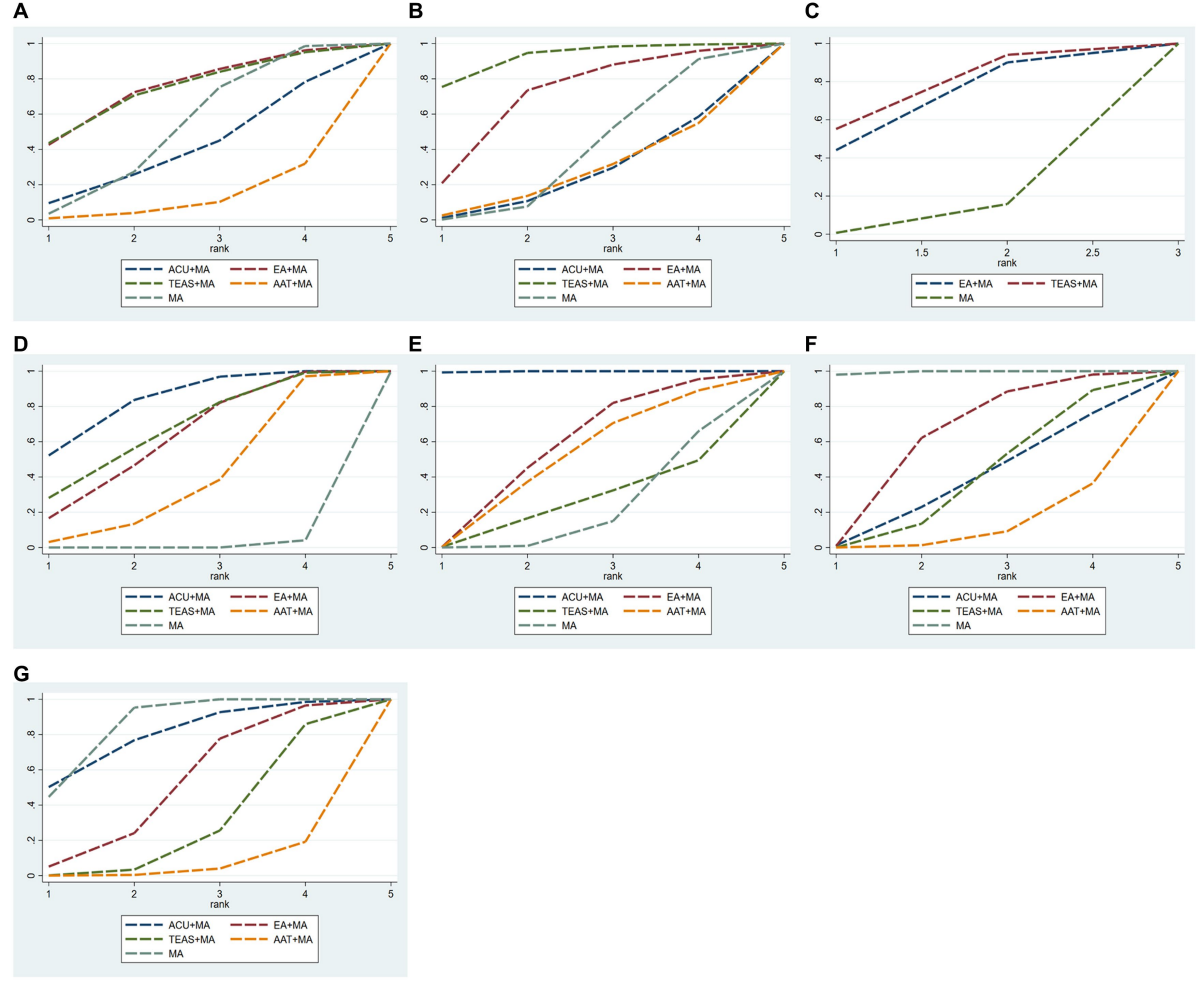
SUCRA probability ranking of interventions. **(A)** VAS-3 scores; **(B)** VAS-7 scores; **(C)** PPT scores; **(D)** HSS scores; **(E)** ROM scores; **(F)** incidence of postoperative nausea and vomiting; **(G)** incidence of postoperative dizziness and sleepiness. ACU, acupuncture; EA, electroacupuncture; TEAS, transcutaneous electrical acupoint stimulation; AAT, auricular acupoint therapy; MA, multimodal analgesia.

(1) As shown in Table 7 and Figure 4A, in terms of improving VAS score on the third day after surgery, the SUCRA probability ranking results showed that EA + MA (SUCRA = 74.2%) > TEAS+MA (SUCRA = 73.3%) > MA (SUCRA = 51.1%) > ACU + MA (SUCRA = 39.7%) > AAT + MA (SUCRA = 11.8%).
(2) As shown in Table 7 and Figure 4B, in terms of improving VAS score on the seventh day after surgery, the SUCRA probability ranking results showed that TEAS+MA (SUCRA = 92.0%) > EA + MA (SUCRA = 69.6%) > MA (SUCRA = 37.8%) > AAT + MA (SUCRA = 25.6%) > ACU + MA (SUCRA = 24.9%).
(3) As shown in Table 7 and Figure 4C, in terms of improving postoperative PPT score, the SUCRA probability ranking results showed that TEAS+MA (SUCRA = 74.4%) > EA + MA (SUCRA = 67.1%) > MA (SUCRA = 8.2%).
(4) As shown in Table 7 and Figure 4D, in terms of improving postoperative HSS score, the SUCRA probability ranking results showed that ACU + MA (SUCRA = 83.2%) > TEAS+MA (SUCRA = 66.5%) > EA + MA (SUCRA = 61.2%) > AAT + MA (SUCRA = 38.0%) > MA (SUCRA = 1.0%).
(5) As shown in Table 4 and Figure 4E, in terms of improving postoperative ROM score, the SUCRA probability ranking results showed that ACU + MA (SUCRA = 99.9%) > EA + MA (SUCRA = 55.9%) > AAT + MA (SUCRA = 49.6%) > TEAS+MA (SUCRA = 24.1%) > MA (SUCRA = 20.5%).
(6) As shown in Table 7 and Figure 4F, the SUCRA probability ranking results for the incidence of postoperative nausea and vomiting were as follows: MA (SUCRA = 99.5%) > EA + MA (SUCRA = 62.4%) > TEAS+MA (SUCRA = 39.0%) > ACU + MA (SUCRA = 37.4%) > AAT + MA (SUCRA = 11.7%).
(7) As indicated in Table 7 and Figure 4G, the SUCRA probability ranking results demonstrate that MA (SUCRA = 85.0%) exhibits a higher incidence of postoperative dizziness and drowsiness compared to ACU + MA (SUCRA = 79.6%), EA + MA (SUCRA = 50.8%), TEAS+MA (SUCRA = 28.7%), and AAT + MA (SUCRA = 5.9%).

**Table 7:** Ranking of SUCRA for each outcome index after TKA using different acupuncture therapy combined with multimodal analgesia.

Treatment	VAS-3		VAS-7		PPT		HSS		ROM		Incidence of PONV		Incidence of PODS	
	Rank (%)	Sort	Rank (%)	Sort	Rank (%)	Sort	Rank (%)	Sort	Rank (%)	Sort	Rank (%)	Sort	Rank (%)	Sort
ACU + MA	39.7	4	24.9	5	_	_	83.2	1	99.9	1	37.4	4	79.6	2
EA + MA	74.2	1	69.6	2	67.1	2	61.2	3	55.9	2	62.4	2	50.8	3
TEAS+MA	73.3	2	92.0	1	74.4	1	66.5	2	24.1	4	39.0	3	28.7	4
AAT + MA	11.8	5	25.6	4	_	_	38.0	4	49.6	3	11.7	5	5.9	5
MA	51.1	3	37.8	3	8.2	3	1.0	5	20.5	5	99.5	1	85.0	1

ACU, acupuncture; EA, electroacupuncture; TEAS, transcutaneous electrical acupoint stimulation; AAT, auricular acupoint therapy; MA, multimodal analgesia; incidence of PONV, incidence of postoperative nausea and vomiting; incidence of PODS, incidence of postoperative dizziness and sleepiness; VAS, visual analog scale; VAS-3, VAS score assessed on the third postoperative day; VAS-7, VAS score assessed on the seventh postoperative day; ROM, the range of motion; HSS, hospital for special surgery; PPT, pain pressure threshold.

#### 3.4.6 Publication bias test

**Figure 5:**
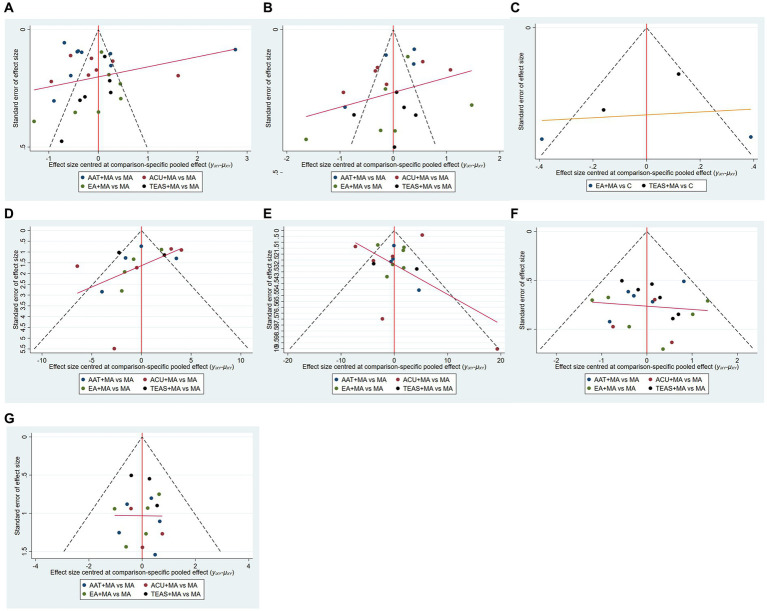
Funnel diagram. **(A)** VAS-3 scores; **(B)** VAS-7 scores; **(C)** PPT scores; **(D)** HSS scores; **(E)** ROM scores; **(F)** incidence of postoperative nausea and vomiting; **(G)** incidence of postoperative dizziness and sleepiness. ACU, acupuncture; EA, electroacupuncture; TEAS, transcutaneous electrical acupoint stimulation.

The adjustment-comparison funnel plots were constructed to assess publication bias for both primary and secondary outcome indicators. However, the asymmetry observed in the HSS score “comparison-adjustment” funnel plots suggests potential publication bias among the included studies. Additionally, some studies examining the primary outcome indicators deviated from the 95% confidence interval of the funnel plot, indicating a possible small sample effect (Figure 5).

## 4 Discussion

This network meta-analysis included 41 randomized controlled trials involving 3,003 patients who underwent TKA. It aimed to explore the effectiveness and safety of different acupuncture therapies in reducing postoperative pain after TKA, evaluating them based on four outcome indicators and the incidence of adverse reactions. The results revealed that EA + MA and TEAS+MA demonstrated superior efficacy in improving the postoperative VAS score of TKA patients. ACU + MA showed better efficacy in enhancing the postoperative ROM score and HSS score of TKA patients. In terms of adverse reactions, AAT + MA effectively reduced the occurrence of postoperative nausea, vomiting, dizziness, and drowsiness. Furthermore, EA + MA, TEAS+MA, and AAT + MA all exhibited better efficacy than MA alone while establishing the safety profile of acupuncture-related therapies for TKA.

The NMA method utilized in this study compared the effectiveness of various acupuncture therapies for postoperative pain and joint function recovery through indirect data analysis, compensating for the lack of direct evidence. These findings provide some support for acupuncture-related treatments to enhance postoperative pain management and facilitate early rehabilitation. In contrast to traditional meta-analyses (95), this study employed the NMA approach to comprehensively assess the included literature and rank different acupuncture therapies. Furthermore, our results confirmed the potential benefits of acupuncture-related interventions in improving postoperative pain and promoting early rehabilitation. Notably, common complications following TKA include nausea, vomiting, dizziness, and drowsiness that can cause significant discomfort for patients. Such symptoms may trigger a self-protective response that makes patients feel seriously ill and leads to reduced activity levels as well as negative attitudes toward early rehabilitation. Therefore, we also evaluated the incidence of adverse reactions after TKA.

The primary objective of TKA is to alleviate the pain associated with knee arthritis, enhance knee joint functionality, and enhance the overall quality of life for patients. However, the occurrence of severe postoperative pain following TKA is a prevalent adverse event that can impede the progress of rehabilitation training. In 1998, Wulf et al. (96) introduced the notion of external focus of attention (EFA), which has garnered significant attention within the realm of rehabilitation medicine (97). The concept of EFA pertains to the process of engaging in actions, specifically skill learning, by directing attention toward the surrounding environment of the action, including sports equipment, the intended goal, and the resultant effect. In contrast, the traditional approach known as Internal Focus of Attention (IFA) centers attention on the action itself, focusing on the involved joints and muscles. EFA aiming at the movement effect has been reported to have more efficacy than IFA aiming at movement characteristics in healthy subjects (98). Research findings have consistently demonstrated that the implementation of EFA, as opposed to IFA, significantly contributes to the restoration of motor function in patients afflicted with anterior cruciate ligament injury (99), ankle sprain (99), stroke (99), and Parkinson’s disease (97). Giacomo Rossettini’s research has revealed that the EFA strategy influences positively the motor performance more than IFA and control and is preferred by the subjects (98). Acupuncture therapy is commonly employed in the rehabilitation of musculoskeletal disorders, including post-total knee arthroplasty. In this study, the acupuncture practitioner primarily instructs patients to perform the EFA strategy, such as attempting to kick a training bat held by the practitioner, as part of the treatment. Postoperative rehabilitation exercise plays a crucial role in mitigating complications, such as knee stiffness and limited activity, for patients undergoing TKA. In clinical practice, it is commonly recommended that patients achieve a knee flexion of 90°and a knee extension of 0°after TKA, to fulfill the general activity requirements (100). The rehabilitation exercise regimen primarily encompasses active knee extension training, straight leg lifting training, and passive knee flexion and extension exercises, with a gradual increment in the angle of knee flexion exercise, while encouraging patients to get out of bed as soon as possible with crutches (101).

The postoperative pain experienced by patients undergoing TKA has detrimental effects on both their physical functioning during exercise and their mental wellbeing. If joint mobility remains restricted and deformities persist following the surgical procedure, it can easily trigger negative emotions. A study conducted by researchers (102) demonstrated a significant correlation (*r* = 0.40, *p* < 0.001) between the severity of depression and the intensity of pain experienced by patients 48 h after the surgery. Postoperative pain is a common adverse event following TKA, with multifaceted causes including mechanical pain resulting from surgical damage to the knee joint and surrounding tissues (103), spasm pain caused by vascular stenosis, tissue ischemia, and edema due to intraoperative tourniquet application (37), neuropathic pain caused by perioperative nerve compression, nerve ischemia, and hypoxia (such as anterolateral knee epidermal nerve injury) (104), and idiopathic pain caused by emotional factors. Although multimodal analgesia schemes have been used in clinical practice for over 20 years to treat post-TKA pain, reports indicate that 10–34% of patients still experience severe pain. Acupuncture has been found to exert peripheral analgesic effects through purinergic signals, the endocannabinoid system, peripheral endogenous opioid peptides, and transient receptor potential vanilloid subfamily (TRPV) channels (36, 105). The reward system and limbic system are related to the central loop of acupuncture-induced pain reduction; among these regions, the rostral anterior cingulate cortex (rACC) is a key brain region. Cannabinoid receptors and dopamine receptors in the brain are closely associated with acupuncture’s ability to relieve pain (106). While different acupuncture therapies operate on floodgate theory mechanisms for their mechanism of action, their afferent effect mechanisms differ. Acupuncture and electroacupuncture regulate dorsal root nerves’ transient potential receptor 1 and purinergic signal P2X3 for analgesic effects (107), while auricular therapy and TEAS may be related to the activation of subcutaneous class C afferent nerve fibers and class A nerve fibers (108). Which afferent effect mechanism is more effective is still unclear. Both acupuncture therapy and exercise therapy have been shown to have a significant therapeutic impact on the rehabilitation process following total knee arthroplasty. The combination of acupuncture therapy and exercise rehabilitation training specifically targets the affected knee joint. Acupuncture therapy effectively alleviates postoperative pain, while exercise therapy promotes active movement and strengthening of the knee joint. The application of acupuncture therapy has been found to enhance local blood circulation, mitigate inflammatory response, and effectively promote detumescence and analgesia. Concurrently, exercise therapy has been shown to enhance the flexion and extension movement function of the knee joint, as well as augment muscle strength (109). The integration of these two treatment modalities effectively addresses the limitations associated with relying solely on a single rehabilitation approach (110).

A meta-analysis study revealed a correlation between pain intensity in musculoskeletal disorders and somatoperception (SoP), space perception (SpP), and body ownership (BO). Following TKA procedures, there was an increase in SoP, SpP, and BO at 3 and 6 weeks post-surgery as pain intensity decreased. Patients with knee osteoarthritis exhibited higher levels of somatosensory dysfunction compared to healthy individuals, while those with fibromyalgia were more susceptible to experiencing bodily illusions. While the precise correlation between pain intensity and the three (SoP, SpP, BO) remains uncertain, it is plausible to posit that the interplay between pain and sensory dysfunction may significantly influence clinical outcomes (111). Previous research (112) has indicated that a majority of individuals diagnosed with knee osteoarthritis (KOA) experience varying levels of proprioception impairments and diminished accuracy. Proprioception is a crucial factor in upholding knee posture and stability during both stationary and active movements as it collaborates with the vestibular and visual systems within a closed loop. Following TKA, patients may restrict their range of motion due to apprehension of pain or unsuitable environmental circumstances, consequently resulting in compromised sensorimotor control functionality. The sensorimotor control disorder of the knee joint, resulting from post-TKA pain, is regarded as a stress response mechanism aimed at reducing further stimulation of painful tissue (113). Over time, this disorder may lead to fatigue and tissue damage in the muscle groups adjacent to the knee joint, exacerbate pain by sensitizing both the peripheral and central nervous systems, and contribute to the disruption of movement patterns (114). Acupuncture is a complex somatosensory stimulation that triggers a wide range of effects in the body (115). Neuroimaging studies have suggested that acupuncture-induced cortical activation mainly reflects the somatosensory, affective, and cognitive processing of pain (116). Genuine acupuncture stimulated several networks of brain activation, including ACC (pregenual anterior, dorsal anterior, and middle), prefrontal cortex, and the parahippocampus (117). The anterior insula is well known for its integrative role in afferent and visceral information and representation of subjective feeling in diverse domains, including pain perception (118). The activation of the ACC also triggers the endogenous analgesia system and modulates sensory transmission at the level of the spinal cord via descending inhibitory modulation (119). The findings of a meta-analysis were that proprioceptive training seemed to alleviate pain and improve the walking speed of patients with knee OA (120). Nevertheless, acupuncture therapy can enhance proprioception of knee joints in patients with osteoarthritis. Simultaneously, acupuncture stimulation increases tactile sensitivity in the ACC and parahippocampus and also strongly influences pain perception in the two-point discrimination task. Acupuncture stimulation leads to the activation of brain regions such as the posterior frontal lobe and anterior cingulate cortex, but the involved interoceptive-autonomic neural network still needs further study (121). Another study concluded that acupuncture may offer analgesic effect that is not dependent on precisely where the needles are inserted so much as that the patient attends to where they are inserted. The mechanism is improvement in self-perception mediated through the sensory discrimination-like qualities of acupuncture (122).

Acupuncture has been found to elicit immune cell stimulation, particularly the activation of regulatory T cells (Treg cells), resulting in increased production of IL − 10 (123). This process also aids in regulating the decrease of macrophages and neutrophils, thereby inhibiting the expression of pro-inflammatory mediators such as IL − 1β, NLRP3, and TNF-α. Consequently, acupuncture demonstrates anti-inflammatory and analgesic effects. Additionally, acupuncture directly activates endogenous opioids, such as enkephalin and β-endorphin, in both the central and peripheral systems, thereby contributing to its analgesic effects (124). In addition to conventional analgesic methods, acupuncture has the potential to alleviate pain indirectly through various mechanisms, including the placebo effect (125), modulation of patients’ negative emotions, and alteration of the contextual effect (CE) (126). The placebo effect (125) associated with sham acupuncture refers to the phenomenon wherein patients do not receive genuine acupuncture treatment, yet experience symptom relief due to their “psychological belief” in the efficacy of the ineffective therapy. The mechanism of the placebo analgesic effect and acupuncture analgesia exhibits similarities. Research employing μ opioid selective tracing and positron emission tomography (PET) to examine the analgesic impact of sham acupuncture demonstrated the presence of endogenous opioids being released in areas associated with pain regulation (127), such as the dorsolateral prefrontal cortex (DLPFC), anterior cingulate cortex (rACC), and periaqueductal gray (PAG). Functional magnetic resonance imaging (fMRI) investigations have additionally demonstrated a decrease in pseudo-needle-induced signals within pain-sensitive cerebral areas, namely, the thalamus and the insular cortex, potentially associated with the alleviation of pain through pseudo-needles (128). Moreover, the placebo response elicited by sham acupuncture involving skin penetration exhibits a greater magnitude of effect compared to sham acupuncture devoid of skin penetration (129). Placebo effect of sham acupuncture incarnates the psychosomatic co-governance of Chinese medicine and also conforms to modern biological–psychological–social–medical philosophy. The anterior cingulate cortex (ACC) serves as the regulatory hub for emotional activity within the limbic system, and it exhibits fiber connections with various brain regions (130). Within the ACC, there exists excitatory glutamate N-methyl-D-aspartate (NMDA), which has garnered considerable interest in the context of pain aversion. Specifically, the NR2 subtype of NMDA is implicated in the modulation of nociceptive perception within the central nervous system and assumes a pivotal role in the development of pain. Acupuncture has been shown to decrease the levels of NR2A and NR2B proteins in the ACC brain region, resulting in a reduction in the excitability of ACC neurons. This downregulation of phosphorylation levels in the ACC contributes to the alleviation of pain aversion and ultimately diminishes the pain experience in patients (131, 132). Contextual factors (CFs) are components of all therapeutic encounters and may constitute the entirety of the perceived effects of the intervention itself or be additive to effects of interventions such as pharmacological and non-pharmacological treatments (133). CFs are perceived cues that affect both the patient and practitioner. Different categories of CFs exist, encompassing patient characteristics, practitioner characteristics, treatment characteristics, the dynamics between the patient and practitioner, and the setting in which the encounter takes place. The size of the observed clinical effects related to CFs can vary significantly based on the patient’s attributes, the practitioner involved, the specific condition being addressed, and the intervention employed. Notably, acupuncture treatment presents a unique challenge in blinding, resulting in a heightened expectation-related placebo effect compared to other placebos (134). For instance, the acupuncturist’s expertise and effective communication foster positive treatment expectations, which frequently correlate with improved treatment outcomes. A significant percentage of postoperative pain relief may be attributed to patients being informed about how the acupuncture therapy will likely impact on their postoperative pain, thus channeling an effective placebo response (135). Expectation-dependent placebo responses are, in principle, mediated by the endogenous opioid system, and the ventromedial prefrontal cortex (vmPFC), the dorsolateral prefrontal cortex (dlPFC), the lateral orbitofrontal cortex (lOFC), hypothalamus, and periaqueductal gray are generally regarded as crucial (136–138). The expectation-dependent placebo effect is similar to acupuncture analgesia, showing that an opioid-dependent cortical network becomes activated during a placebo response. The challenge of managing patients’ expectations in musculoskeletal pain remains a significant issue. Clinicians must carefully determine the appropriate timing and approach to address negative expectations, weighing the potential benefits of minimizing nocebo effects against the potential risks of jeopardizing the therapeutic alliance and increasing drop-out rates. Clinicians may use the intensity of patients’ expectations as a gauge to determine whether a direct challenge (i.e., optimization) or an indirect challenge of their beliefs (i.e., violation) is more appropriate, to preserve the therapeutic alliance and avoid damages (139).

Currently, total knee arthroplasty stands as the most efficacious intervention for advanced rheumatoid arthritis (RA), a prevalent inflammatory arthropathy (IA). It is noteworthy that a significant proportion of patients undergoing total knee arthroplasty for advanced RA present with comorbidities. A meta-analysis (140) reveals a substantial rise in the prevalence of fibromyalgia (FM) among individuals with inflammatory arthritis, with rates reaching 18–24% in those with RA. FM is typified by widespread and enduring musculoskeletal pain, frequently accompanied by psychological conditions such as anxiety and depression (141). The studies have found objective alterations or dysfunctions of the large Aβ and of the group of thin Aδ and C fibers (the so-called “small-fibers”) in approximately 50% of FM patients (142). It is believed that the analgesic effects of electroacupuncture and transcutaneous electrical acupoint stimulation are mediated by the activation of these nerve fibers. This network meta-analysis demonstrates that acupoints, including Sanyinjiao (SP06), Zusanli (ST36), Hegu (LI04), and Taichong (LR03), exhibit potential for pain management following TKA. Specifically, acupuncture at Sanyinjiao exhibits the ability to mitigate hyperactivity within the hypothalamic–pituitary–adrenal (HPA) axis by reducing serum levels of norepinephrine and cortisol, thereby ameliorating non-specific symptoms associated with FM, such as insomnia, anxiety, and depression (143). Furthermore, acupuncture at Zusanli exhibits potential in the treatment of rheumatic immune diseases through immunoregulatory mechanisms and the inhibition of inflammatory cytokine release (144). Acupuncture administered at the Hegu acupoint has been found to stimulate the release of plasma β-endorphin, regulate the activity of the hypothalamic–pituitary system, and suppress both pain perception and emotional response (145). Similarly, acupuncture at the Taichong acupoint has been observed to induce alterations in brain activation signals and provide symptomatic relief for emotional and pain-related disorders (146). Consequently, acupuncture therapy not only effectively addresses postoperative pain and enhances knee function following TKA but also demonstrates clinical efficacy in the treatment of fibromyalgia, potentially exhibiting multiple therapeutic effects.

There are still some limitations in this study: (1) As a result of the distinctive characteristics inherent in acupuncture practice, certain studies failed to furnish comprehensive details regarding the specific randomization and blinding techniques employed during implementation. Since acupuncture operations cannot be blinded to patients, the principle of triple separation of clinical operators, efficacy evaluators, and statistical analysts can be utilized. (2) The risk of bias assessment of the included studies was mostly unclear, and the quality of the literature was low. Therefore, in future studies, reporting should be based on the Consolidated Standards of Reporting Trials (CONSORT) to ascertain the literature quality (147). (3) The absence of any analgesic regimen during the perioperative period for patients undergoing TKA contradicts the principles of ERAS. Consequently, a majority of the studies included in this analysis were semi-randomized controlled trials, which lacked placebo control groups. There exist variations in the parameters of EA and TEAS across different studies. Acupuncture therapy has many differences in the acupoint selection, manipulation, and frequency, which could cause heterogeneity. Well-designed subgroup analysis should have been conducted. (4) Furthermore, a majority of the studies included in the analysis were small-scale randomized controlled trials, which may introduce selection bias and implementation bias, consequently resulting in low quality of literature. (5) Due to limitations within the original literature, intervention measures were categorized into ACU + MA, EA + MA, TEAS+MA, AAT + MA, and MA interventions. The results suggest that combined therapies have better performance in improving the outcome indicators. However, there are many combinations of combined therapies, which will be further elaborated in subsequent studies. (6) The assessment of PPT was conducted using a restricted number of studies, potentially leading to biased outcomes. (7) The studies included in the analysis primarily emphasized the short-term effectiveness of acupuncture therapy in terms of alleviating postoperative pain and rehabilitating joint function after TKA surgery. However, the evaluation of long-term efficacy for acupuncture therapy remains inconclusive. Therefore, the overall effectiveness of acupuncture therapy in treating post-TKA pain warrants further scrutiny. (8) The majority of the studies included in this review were published in Chinese journals, which introduce a potential publication bias associated with Chinese culture.

In future research, a more detailed examination of various acupuncture therapies should be prioritized by researchers. While numerous studies have examined the mechanism and clinical outcomes of acupuncture in treating postoperative pain following TKA, there is a lack of exploration regarding the efficacy of varying acupoint selection, acupuncture depth, needle retention time, and treatment frequency. Future clinical research should prioritize investigating the regularity of acupoint selection and acupuncture methods in effectively managing postoperative pain in TKA patients and provide theoretical basis of acupuncture for the clinical treatment of postoperative pain. Furthermore, a variety of studies have explored the mechanisms of acupuncture, particularly focusing on signaling pathways and cytokines. However, the majority of studies examining signaling pathways have primarily utilized nodal signaling pathways as their subjects, resulting in a lack of coherence and comprehensiveness. Moreover, the precise signaling pathways underlying the effectiveness of acupuncture in managing postoperative pain following TKA remain inadequately understood, highlighting the necessity for expanded and comprehensive research efforts. Future investigations should integrate proteomics and genomics principles and methodologies to elucidate the specific regulatory mechanisms and distinctive features of acupuncture in TKA postoperative pain management, as well as find new clues for the treatment of TKA postoperative pain through animal studies of acupuncture.

## 5 Conclusion

The limitations of this study underscore the necessity for future clinical practice to address the following aspects: achieving consensus among clinicians and researchers to harmonize inclusion criteria for patient recruitment, designing study protocols and reporting results in strict adherence to the CONSORT principle, and registering study protocols on reputable registration platforms prior to trial commencement. Additional well-designed, large-sample, multi-centric clinical trials and comprehensive meta-analyses are necessary to further substantiate and enhance the validity of our results.

In general, different acupuncture therapies have advantages in terms of efficacy, safety, and patient compliance. EA + MA and TEAS+MA may be considered as optimal choices for mitigating postoperative pain in patients undergoing TKA, while ACU + MA may be preferable for enhancing postoperative knee function. AAT + MA could be regarded as an effective option for preventing postoperative nausea and vomiting, as well as dizziness and drowsiness. Moreover, the combination of acupuncture-related therapies with multimodal analgesia demonstrates significant overall benefits and safety, thus suggesting the utilization of these therapies in clinical settings.

## Data availability statement

The original contributions presented in the study are included in the article/Supplementary material, further inquiries can be directed to the corresponding author.

## Author contributions

NL: Conceptualization, Data curation, Formal analysis, Resources, Software, Writing – original draft, Writing – review & editing. GL: Data curation, Visualization, Writing – review & editing. XC: Data curation, Formal analysis, Funding acquisition, Supervision, Writing – review & editing. YX: Investigation, Resources, Writing – review & editing. YH: Conceptualization, Data curation, Formal analysis, Software, Writing – review & editing. DZ: Methodology, Software, Writing – review & editing. LW: Resources, Supervision, Writing – review & editing. SC: Conceptualization, Funding acquisition, Methodology, Writing – review & editing.
