# Supplementary material for: Combining various acupuncture therapies with multimodal analgesia to enhance postoperative pain management following total knee arthroplasty: a network meta-analysis of randomized controlled trials
Source: Front Neurol. 2024 Mar 18;15:1361037. doi: 10.3389/fneur.2024.1361037 (PMC10984270; doi:10.3389/fneur.2024.1361037)
Supplement: Supplementary file 2 [file Data_Sheet_2.docx]

**Appendix 2. The search strategies for the included RCTs for Pubmed.**

#1 Acupuncture[MeSH Terms]

#2 Acupuncture Points[MeSH Terms]

#3 Acupuncture, Ear[MeSH Terms]

#4 Acupuncture Analgesia[MeSH Terms]

#5 Acupuncture Therapy[MeSH Terms]

#6 Auriculotherapy[MeSH Terms]

#7 Acupuncture*[Title/Abstract] OR Needling[Title/Abstract] OR Electroacupuncture* [Title/Abstract] OR Electro-acupuncture [Title/Abstract] OR Transcutaneous electrical stimulation of acupoints [Title/Abstract] OR Acupoint Therapy[Title/Abstract] OR Acupuncture Treatment[Title/Abstract] OR Acupuncture Treatments[Title/Abstract] OR Needle Therapy[Title/Abstract] OR silver needle[Title/Abstract] OR moxibustion [Title/Abstract] OR de qi[Title/Abstract] OR meridian [Title/Abstract] OR Auriculotherapy[Title/Abstract] OR needle pricking[Title/Abstract] OR needling[Title/Abstract] OR fire needle[Title/Abstract] OR fire needling[Title/Abstract] OR three-edged needle[Title/Abstract] OR blood letting Therapy [Title/Abstract] OR pricking blood therapy[Title/Abstract] OR Needle Warming Therapy[Title/Abstract] OR scalp acupuncture[Title/Abstract] OR auricular acupuncture[Title/Abstract] OR ear acupuncture[Title/Abstract] OR intradermal needling[Title/Abstract] OR acupoint embedding therapy[Title/Abstract]

#8 #1 OR #2 OR #3 OR #4 OR #5 OR #6 OR #7

#9 total knee replacement[MeSH Terms]

#10 total knee replacement [Title/Abstract] OR total knee replacement*[Title/Abstract] OR total knee arthroplasty[Title/Abstract] OR knee surgery[Title/Abstract] OR TKA[Title/Abstract]

#11 #9 OR #10

#12 Randomized Controlled Trial [Publication Type]

#13 Randomized Controlled Trials as Topic[MeSH Terms]

#14 Randomized Controlled Trial[All Fields] OR random*[Title/Abstract] OR RCT[All Fields] OR Trial*[All Fields]

#15 #12 OR #13 OR #14 OR

#16 #8 AND #11 AND #15

**Appendix 2. The search strategies for the included RCTs for EMBASE.**

#1 'Acupuncture'/exp

#2 'Acupuncture Points'/exp

#3 'Acupuncture, Ear'/exp

#4 'Acupuncture Analgesia'/exp

#5 'Acupuncture Therapy'/exp

#6 'Auriculotherapy'/exp

#7 'acupuncture*':ti,ab OR 'electroacupuncture*':ti,ab OR 'electro-acupuncture':ti,ab OR 'acupoint therapy':ti,ab OR 'transcutaneous electrical stimulation of acupoints*':ti,ab OR 'Acupuncture Treatment':ti,ab OR 'Acupuncture Treatments':ti,ab OR 'needle therapy':ti,ab OR 'silver needle':ti,ab OR 'moxibustion':ti,ab OR 'de qi':ti,ab OR 'meridian':ti,ab OR 'auriculotherapy':ti,ab OR 'needle pricking':ti,ab OR 'needling':ti,ab OR 'fire needle':ti,ab OR 'fire needling':ti,ab OR 'three-edged needle':ti,ab OR 'blood letting therapy':ti,ab OR 'pricking blood therapy':ti,ab OR 'needle warming therapy':ti,ab OR 'scalp acupuncture':ti,ab OR 'auricular acupuncture':ti,ab OR 'ear acupuncture':ti,ab OR 'intradermal needling':ti,ab OR 'acupoint embedding therapy':ti,ab

#8 #1 OR #2 OR #3 OR #4 OR #5 OR #6 OR #7

#9 ' total knee arthroplasty '/exp

#10 ' total knee arthroplasty ':ti,ab OR ' total knee arthroplasty*':ti,ab OR ' total knee replacement':ti,ab OR ' knee surgery ':ti,ab OR ' TKA ':ti,ab

#11 #9 OR #10

#12 'randomized controlled trial'/exp

#13 'randomized controlled trial (topic)'/exp

#14 random*:ti,ab OR random*:ti,ab OR 'RCT':ti,ab OR Trial*:ti,ab

#15 #12 OR #13 OR #14

#16 #8 AND #11 AND #15

**Appendix 2. The search strategies for the included RCTs for Cochrane Library**

#1 MeSH descriptor: [Acupuncture] explode all trees

#2 MeSH descriptor: [Acupuncture Points] explode all trees

#3 MeSH descriptor: [Acupuncture, Ear] explode all trees

#4 MeSH descriptor: [Acupuncture Analgesia] explode all trees

#5 MeSH descriptor: [Acupuncture Therapy] explode all trees

#6 MeSH descriptor: [Auriculotherapy] explode all trees

#7 'acupuncture*':ti,ab OR 'electroacupuncture*':ti,ab OR 'electro-acupuncture':ti,ab OR 'transcutaneous electrical stimulation of acupoints*':ti,ab OR 'acupuncture*':ti,ab OR 'electroacupuncture*':ti,ab OR 'electro-acupuncture':ti,ab OR 'acupoint therapy':ti,ab OR 'Acupuncture Treatment':ti,ab OR 'Acupuncture Treatments':ti,ab OR 'needle therapy':ti,ab OR 'silver needle':ti,ab OR 'moxibustion':ti,ab OR 'de qi':ti,ab OR 'meridian':ti,ab OR 'auriculotherapy':ti,ab OR 'needle pricking':ti,ab OR 'needling':ti,ab OR 'fire needle':ti,ab OR 'fire needling':ti,ab OR 'needle warming therapy':ti,ab OR 'scalp acupuncture':ti,ab OR 'auricular acupuncture':ti,ab OR 'ear acupuncture':ti,ab OR 'intradermal needling':ti,ab OR 'acupoint embedding therapy':ti,ab

#8 #1 OR #2 OR #3 OR #4 OR #5 OR #6 OR #7

#9 MeSH descriptor: [total knee arthroplasty] explode all trees

#10 'total knee arthroplasty':ti,ab OR 'total knee arthroplasty*':ti,ab OR ' total knee replacement ':ti,ab OR ' knee surgery ':ti,ab OR ' TKA ':ti,ab

#11 #9 OR #10

#12 MeSH descriptor: [Randomized Controlled Trial] explode all trees

#13 MeSH descriptor: [Randomized Controlled Trials as Topic] explode all trees

#14 random*:ti,ab OR random*:ti,ab OR 'RCT':ti,ab OR Trial*:ti,ab

#15 #12 OR #13 OR #14

#16 #8 AND #11 AND #15

**Appendix 2. The search strategies for the included RCTs for** **China National Knowledge Infrastructure (CNKI)**

SU='针灸'+'针刺'+'电针'+'温针’+‘温针灸'+'艾灸'+'灸法'+'耳穴'+'耳针'+'体针'+'头针'+'火针'+'梅花针'+'穴位埋线'+'穴位敷贴' AND SU=('全膝关节置换术'+'TKA' ) AND FT='随机'

**Appendix 2. The search strategies for the included RCTs for Wanfang Data**

题名或关键词:(针灸 or 针刺 or 电针 or 温针 or 温针灸 or 艾灸 or 灸法 or 耳穴 or 耳针 or 体针 or 头针 or 火针 or 梅花针 or 穴位埋线 or 穴位敷贴) and 题名或关键词:(全膝关节置换术 or TKA) and 全部:(随机)

**Appendix 2. The search strategies for the included RCTs for** **Chinese Scientific Journal Database (VIP)**

M=(针灸 OR 针刺 OR 电针 OR 温针 OR 温针灸 OR 艾灸 OR 灸法 OR 耳穴 OR 耳针 OR 体针 OR 头针 OR 火针 OR 梅花针 OR 穴位埋线 OR 穴位敷贴) AND M=(全膝关节置换术 OR TKA) AND (U=随机)

**Appendix 2. The search strategies for the included RCTs for China Biology Medicine (CBM)**

("针灸"[常用字段:智能] OR "针刺"[常用字段:智能] OR "电针"[常用字段:智能] OR "温针"[常用字段:智能] OR "温针灸"[常用字段:智能] OR "艾灸"[常用字段:智能] OR "灸法"[常用字段:智能] OR "耳穴"[常用字段:智能] OR "耳针"[常用字段:智能] OR "体针"[常用字段:智能] OR "头针"[常用字段:智能] OR "火针"[常用字段:智能] OR "梅花针"[常用字段:智能] OR "穴位埋线"[常用字段:智能] OR "穴位敷贴"[常用字段:智能]) AND ("全膝关节置换术"[常用字段:智能] OR "TKA"AND"疼痛"[常用字段:智能] AND ("随机"[全部字段:智能] OR "随机对照试验（主题）"[不加权:扩展] OR "随机对照试验"[不加权:扩展])
